# Comprehensive Literature Review on Metal Nanoparticle for Enhanced Shelf Life of Mango Fruit

**DOI:** 10.1155/2024/4782328

**Published:** 2024-06-24

**Authors:** Yalew Yiblet, Indiris Abdu, Basaznew Belew

**Affiliations:** ^1^ Department of Biology Mekdela Amba University, P.O. Box 32, Tulu Awlia, Ethiopia; ^2^ Department of Mathematics Mekdela Amba University, P.O. Box 32, Tulu Awlia, Ethiopia

## Abstract

The purpose of this review was to investigate the application of metal nanoparticles in fruit shelf life extension. Despite growing interest in nanoparticles and their potential applications, there are currently few effective methods for prolonging the shelf life of fruits. The study concentrated on the principles underlying the shelf life extension of metallic nanoparticles, including copper oxide, zinc oxide, silver, and titanium oxide. The biological properties of nanoparticles, especially those with antibacterial qualities, have drawn interest as possible fruit preservation solutions. Many conventional preservation methods have drawbacks, including expensive production costs, short shelf lives, undesirable residues, and the incapacity to properly keep perishable fruits in their natural environments. Techniques for extending shelf life based on nanotechnology have the potential to get around these problems. The review focused on the effective use of environmentally benign, green synthesis-produced nanoparticles to extend the fruit shelf life. The ability of these nanoparticles to successfully preserve fresh fruits was established. The results imply that fruit preservation by the use of nanoparticle synthesis techniques may be a viable strategy, offering a more effective and sustainable substitute for traditional procedures.

## 1. Introduction

Mango (*Mangifera indica* L.) is a fruit crop of utmost significance in tropical and subtropical regions. In the period of rapidly changing climates, farmers, especially smallholders, have demonstrated a strong interest in diversifying their agricultural produce to include fruits like mangoes and setting profit-making goals that help achieve food security [1]. The rising popularity of mangoes can be attributed to shifting consumer preferences [2]. The acceptance of mango fruit is strongly associated with both internal and external quality parameters, which are primarily influenced by the flavor of sugars, acids, and volatile components that give the fruit a distinctive taste [3]. High postharvest and marketing chain losses and a shorter storage period are caused by improper postharvest handling, disease incidence, and sensitivity to chilling injury [4]. The handling and transportation of this fruit require proper handling. Mango preservation methods are explored in the literature in a variety of ways to prolong shelf life and maintain their organoleptic and nutritional qualities. These methods include refrigeration, modified atmosphere packaging, controlled atmosphere, and traditional methods [5]. An estimated 55–60 million metric tons of mangoes are produced worldwide each year. India, China, Thailand, Mexico, and Indonesia are the five leading countries in the global mango market, together producing around 77% of all mangoes produced annually [6].

Poor storing practices damage about 5% of the world's fruit crop. In developing countries, improper handling and storage methods can result in over 50% of mango fruit being lost after harvest due to physiological and pathological problems [7]. Mango fruit is taken from the tree and ripens quickly in 3 to 9 days at room temperature. This weakens the fruit's tissue and increases its susceptibility to microbial infection and mechanical injury [8].

Nanotechnology is an area of study that studies the design, manufacture, and characterisation of a particle of matter with a diameter of 1 to 100 nanometers (nm) and is called an ultrafine or nanoparticle [9]. This term is sometimes also used to express fibers and tubes that are smaller than 100 nm or larger particles up to 500 nm. Due to their special physical, chemical, and antibacterial qualities, nanoparticles can be utilized to preserve fruit [10]. The use of coated fruit loaded with metallic nanoparticles has recently become a guarantee of a safe way of preserving mango fruit. Metal oxide nanoparticles (NPs) can be made using a variety of techniques, such as chemical, physical, and biological (or “green synthesis”) approaches [11]. NPs produced by physical and chemical processes have abnormal characteristics, including high water repellency, which makes them unsuitable for biological uses. But the naturally occurring reducing and biotemplating chemicals in greenly produced NPs also contribute to the priming or functionalization [12].

Recent years have seen a great deal of research on the potential antibacterial properties of metallic nanoparticles [13]. The findings of the microbiological tests can offer important information on how well the nanopackaging coatings work to improve microbiological safety and lengthen the shelf life of packed mangoes fruit [14].

Nanoparticles have a variety of shapes [15]. Shapes might be spherical, hexagonal, rod, crystalline, needle, amorphous, cube, or triangle [16]. As a consequence of their spherical shape and high surface area-to-volume ratio, these particles have a wide range of potential applications [17]. To solve this problem and provide information to researchers, the fruit industry, fruit consumers, and stalk holders, this study focused on the enhancement of the shelf life of fruit using metallic NPs. It was designed to draw on previously published efforts in this field of study.

## 2. Methodology

A comprehensive search and collection of research publications was conducted from various databases with an emphasis on the application of nanoparticles (NPs) to prolong the shelf life of mango fruits. The objective of the compilation was to create an extensive database of research on the use of NPs to maintain the freshness and quality of mango fruits. Since many published studies were only accessible independently, a careful analysis was necessary to bring the data together and reach a definitive result while minimizing the likelihood of conflicting information, ambiguities, or misinterpretations. The review aimed to emphasize the synthesis process, the characterisation method, particle size, antibacterial mechanisms, and the key conclusion. It was conducted in accordance with existing systematic reviews. This review adhered closely to the criteria and guidelines provided by PRISMA-2020 (Reporting Items for Systematic Reviews and Meta-Analyses) to ensure methodological rigor and transparency [18].

### 2.1. Formulation of Research Questions and Problems

The question “What are the mechanisms of metallic nanoparticles in extending the shelf life of mango fruits?” provided the conceptual basis for this methodical investigation. When the importance of nanoparticles in the contemporary world was examined and assessed, the problem was discovered. As a result of its diverse relevance, the study focuses on studying the shelf-life activity of metallic nanoparticles.

### 2.2. Search Engine for Research Articles

**Figure 1:**
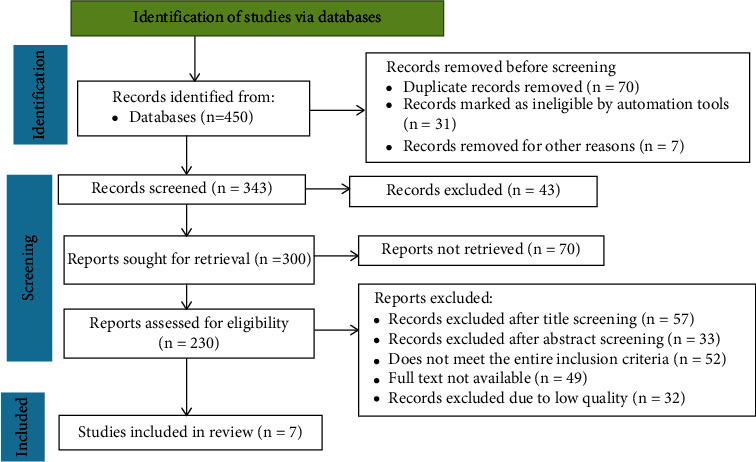
PRISMA-2020 flow diagram of eligible studies.

An extensive search was conducted across a number of databases and registers, including PubMed, Science Direct, Web of Science, Google Scholar, and the Directory of Open Access Journals, in order to compile a wide variety of research publications. Other resources, including websites, respectable organizations, and citations, were also examined in addition to these academic platforms. The following important words and phrases were taken from the research publications' titles, abstracts, and keywords. To search for the terms “Nanoparticles,” “Metal Nanoparticles,” “Metal Oxide Nanoparticles,” “Shelf Life of Fruit,” and “Mechanisms of Action” independently or in combination, Boolean operators (“OR” or “AND”) were utilized. The study was conducted from October 2023 to March 2024. The search process, the items that were included and removed, and the justifications for the exclusions were presented in accordance with the PRISMA-2020 flow diagram criteria [18] (Figure 1).

### 2.3. Inclusion and Exclusion Criteria for Included Studies

#### 2.3.1. Inclusion Criteria

- Original research articles, focused on the utilization of metallic nanoparticles to extend the shelf life of fruits by preventing spoilage caused by microorganisms, were searched.
- Design of experimental studies.
- To ensure that the study encompassed the most up-to-date and accessible information, recent studies published in English and available for free online were included in the research. Using studies published in English, the study aimed to access a wide range of relevant and current scientific literature studies.

#### 2.3.2. Exclusion Criteria

- The results related to other metallic nanoparticles and their functions are not connected to the fruit shelf life.
- Research that has been published in other languages without peer review.
- Articles that have undergone a previous review, poor-quality publications, and publications that are duplicates or analyses that are expanded upon from the initial study.

### 2.4. Data Extraction

A data abstraction technique was used to create the data for every article that was included. This procedure guaranteed consistency in data collection and made it possible to systematically extract pertinent information from the articles. The data extraction process included metallic nanoparticles, the synthesis method, characterisation, particle size, findings, and references, among other important components (Table 1).

### 2.5. Quality Assessment of Each Included Study

For assessing the quality of data presentation, validity, and reliability collected from included publications in a systematic review, the PRISMA 2020 checklist item is a great resource [18]. A standardized framework for performing and reporting systematic reviews is provided by the PRISMA checklist (Preferred Reporting Items for Systematic Reviews and Meta-Analyses), assuring rigor and transparency in the review process. To evaluate the overall quality of the evidence in the study, the grading of recommendation evaluation, development, and evaluation (GRADE) approach was employed. Three main assessment criteria were used to assess each study's quality: methodological quality, comparability, study outcome, and statistical analysis [19]. These criteria are essential for evaluating the validity and reliability of the study results. High-quality publications earned four to five points, moderate-quality papers received three points, and low-quality papers received zero to two points. The choice and evaluation of quality were independently performed by three reviewers (YY, IA, and BB). The articles were added after an agreement was reached, and the discrepancies between the reviewers were resolved through discussion. Each of the four reviewers (YY, IA, and BB) made their own independent decisions and assessed quality. After reaching a consensus, the articles were included and the disagreements of the reviewers were settled through dialogue.

## 3. Results

### 3.1. Findings from the Literature Search

**Figure 2:**
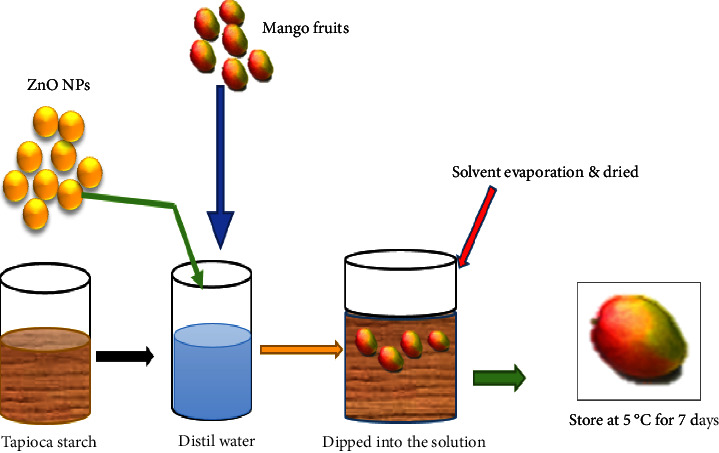
Extending the shelf life of mango fruit using ZnO nanoparticles.

Table 1 shows that all of the included research addressed metallic and metallic oxide nanoparticles and showed how to stop microbial spoiling, which leads to fruit rotting, in order to extend the fruit's shelf life. Nanoparticles have been studied as potential fruit shelf life extenders due to their unique properties. One way is to coat the fruit's surface with edible coatings that contain nanoparticles, like ZnO nanoparticles, to provide a protective layer (Figure 2). These investigations focused on the action of metallic NPs and their potential benefits in prolonging the freshness and quality of mango fruits.

In this instance, the goal of using metallic NPs is to extend the shelf life of mangoes and decrease postharvest losses. By incorporating metallic nanoparticles (NPs) into appropriate packaging or treatment techniques, researchers have explored the potential of NPs to suppress microbial development, delay the ripening process, and maintain the overall quality of mango fruits. The utilization of metallic nanoparticles may be able to increase the shelf life of mango fruits, based on the findings of these studies (Table 1).

### 3.2. Characteristics of the Eligible Studies

Worldwide, there have been about 450 papers on the application of nanoparticles in fruit packing and preservation. The records that were removed consisted of seven records (*n* = 7), duplicate records (*n* = 31), and records that were deemed ineligible by automated tools (*n* = 70). There were 300 articles available for retrieval and other techniques after 43 articles were further eliminated following the first screening. Seventy of these papers, however, were not retrieved for additional examination. Based on the preset inclusion and exclusion criteria, a total of 223 articles were eliminated following close inspection and analysis. After a thorough screening process, only seven reports were found to be eligible for inclusion in the final study, which is illustrated in Figure 1.

### 3.3. Mechanism of Shelf-Time Enhancement with NPs

Nanoparticles (NPs) can prolong the shelf life of fruits using a complex process that considers several parameters. They possess antibacterial properties similar to silver nanoparticles. They have the power to halt the development of bacteria, fungi, and other microorganisms that contaminate the fruit. These nanoparticles can disrupt microbial cell membranes or interfere with cell processes, reducing microbial activity and extending fruit shelf life (Table 1). Nanoparticle-containing films or nanocoatings can alter fruit surface characteristics, alter gas barrier characteristics, and control gas flow between the fruit and its surroundings. This helps preserve ideal gas concentrations, increase fresh produce's shelf life, and improve preservation [20]. By controlling gas permeability, postponing ripening and senescence, and extending the fruit's shelf life, the rate of respiration and ethylene production can be decreased (Figure 2).

### 3.4. Synthesis of Nanoparticles

**Figure 3:**
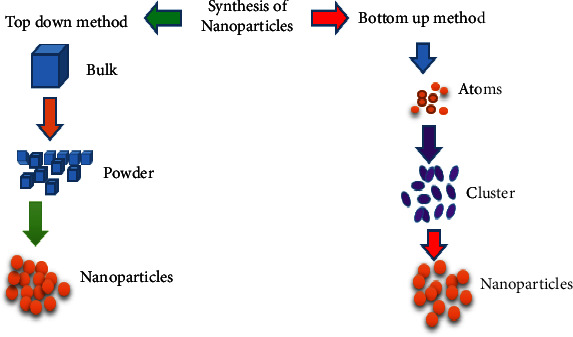
Top-down and bottom-up synthesis techniques of nanoparticles.

**Figure 4:**
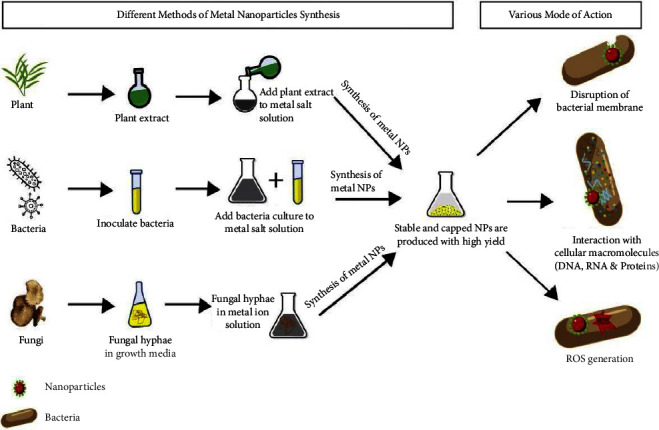
Metal nanoparticles produced from plants, microbes, and fungi in an environmentally friendly manner (created by Verma et al. [21]).

One can distinguish between two broad categories of synthesis: top-down synthesis and bottom-up synthesis. These methods explain various approaches of producing nanoparticles based on size reduction or assembly. Synthesis from the top down, which is the process of producing nanoparticles, involves the reduction of bulk material's size (Figure 3). Additionally, “green synthesis” refers to the environmentally acceptable process of producing metal nanoparticles using natural, nontoxic, and sustainable substances (Figure 4).

## 4. Discussion

The short shelf life of fruits can have a variety of negative implications on the economy and environment, such as decreased market accessibility, food loss and waste, and financial losses for producers and retailers. In the fruit industry, a variety of antimicrobial substances are still used to extend the shelf life of fruits, as well as to preserve and disinfect fruit and food products. In order to combat the short shelf life of fruits, new technology with different mechanisms of action must be developed. At the present time, more research is being done on the ability of metallic NPs (Ag, ZnO, CuO, and TiO_2_) to extend the shelf life of fruit. Knowing several ways nanoparticles could prolong the shelf life of fruits can help buyers, sellers, and other stakeholders make well-informed decisions.

### 4.1. Mechanism of Fruit Shelf Life Enhancement Using Metallic NPs

As a result of removing bacteria that cause fruit deterioration, NPs have demonstrated a major impact on extending the shelf life of fruits. Numerous investigations have shown antimicrobial characteristics of metallic nanoparticles and their capacity to regulate microbial proliferation in fruits. The antimicrobial effects of silver nanoparticles (AgNP) in fruit conservation have been highlighted by Ali et al. [22], Elatafi and Fang [23], and Ferrone [24]. Similarly, Laurenti and Cauda [25], Qi et al. [26], Li et al. [27], Rahisuddin et al. [28], Singh et al. [29], Ali et al. [30], Lallo et al. [31], Juan et al. [32], and Patel et al. [33] showed that ZnO prolongs the shelf life of the fruit in a variety of ways. Its antibacterial activity, which aids in preventing the growth of bacteria that cause spoiling on the fruit's surface, is one of the main mechanisms. ZnO also functions as a barrier, stopping the flow of gases that cause ripening and fruit degradation, such as ethylene and oxygen. This barrier effect prolongs the firmness, color, and general quality of the fruit. Furthermore, it has been demonstrated by Salunkhe et al. [34], Ramadan and Moersel [35], Cvanić et al. [36], Sharma et al. [37], and Alizadeh Sani et al. [38] that treating fruits with copper oxide nanoparticles (CuO) prolongs their shelf life and maintains their quality. The fact that titanium oxide nanoparticles (TiO_2_) prolong the shelf life of fruits has also been shown in numerous studies. Studies conducted by Yong and Liu [39], Jiang et al. [40], Fathi et al. [41], Ghosh et al. [42], and Patel and Mishra [43] are a few of these studies. Antimicrobial agents can be added to food packaging materials to suppress bacteria and extend the product's realistic useable life. Various metal and metal oxide nanoparticles have antimicrobial properties and can be used in fruit packaging [44].

### 4.2. Method for Nanoparticle Synthesis

Compared to other chemical or conventional methods, green synthesis is a less hazardous method of producing desirable nanoparticles when natural strains and plant extracts are used as reducing and capping agents [45]. The following reasons are listed in the answer: unique properties, minimal risk and toxicity, decreased surface imperfection, affordability, and ease of access [46]. As a result, the green or biological production of nanoparticles presents an alluring method of doing so and promises to assist in resolving these chemical and physical issues, hence reducing the risks to the environment [47]. Numerous elements, including low synthesis costs, quick development durations, accessibility, environmental friendliness, the possibility of high manufacturing volumes, and the usage of plant-based components, can be credited for this [48].

### 4.3. Effect of Nanoparticles' Shape and Size

According to Seil and Webster [49], the small size of nanoparticles facilitates their entry into microbial cells, hence enhancing their interaction and contact with bacteria. The surface area to volume ratio of a nanoparticle is particularly important since a higher surface area may result in increased contact with microbial cells, which may compromise structural and functional aspects of the particle [50]. As stated by Duncan [51], their sizes and forms might affect how effective they are against dangerous microbes. AgNPs with particle sizes between 1 and 10 nm have been shown to have the strongest antibacterial effects by directly interacting with bacterial cell walls and membranes, which causes splinters and holes to form, less sugar leakage, and ultimately bacterial death [52].

### 4.4. Future Perspectives

The results of this study could provide insight into potential future advancements that could improve their antibacterial and physical properties for practical use. In order to ascertain the necessary conditions for their production and application, future research ought to concentrate on enhancing the consistency of composite-coating attributes and tracking their impact on the fruits and vegetable storage quality [53]. In the upcoming years, the utilization of silver nanoparticles may have a significant positive impact on the development of polymeric materials for active food packaging [54]. Nonetheless, the primary concern regarding the application of these kinds of packaging materials is the lack of clarity regarding the toxicity and safety of silver nanoparticles [55]. Thus far, studies have demonstrated that mammalian cells can become toxically affected by silver nanoparticles. In human cells, for instance, AgNPs cause cytotoxicity, genotoxicity, and an inflammatory response [56]. AgNP capping may show tremendous promise for usage as a fungicidal and bactericidal agent, and it may also lessen or even eliminate cytotoxic effects. Skin, respiratory, and gastrointestinal contact are the risk pathways for nanomaterial exposure [57]. Given the potential for nanoparticles to migrate from the nanocomposites to the food surface, it is apparent that the main concern posed by nanocomposite materials employed in food packaging is related to human ingestion [58]. Therefore, in order to figure out the dangers associated with human health, a thorough toxicological investigation is required. Human safety seems to be one of the primary considerations when employing nanoparticles as food packaging materials because concerns about their health and safety are still poorly understood. The maximum number of nanoparticles that can be present in food must be established in order to shield consumers from risk exposure that they did not choose. The biggest obstacle that researchers are currently encountering seems to be establishing such regulatory criteria [59].

## 5. Conclusions

In conclusion, it has been found that using metallic nanoparticles to preserve the quality and extend the shelf life of mango fruits during postharvest storage and transit is a plausible strategy. Antimicrobial and antioxidant capabilities of metallic nanoparticles, such as titanium dioxide (TiO_2_·NPs), zinc oxide (ZnO·NPs), copper oxide (CuO·NPs), and silver (AgNPs), have been shown to be efficient in preventing the growth of spoilage microorganisms and delaying the ripening and senescence processes in mangoes. It has been demonstrated that, depending on the kind and concentration of these metallic nanoparticles, applying them as coatings or in packing materials can keep mangoes fresher longer than they would under normal storage circumstances. Mangoes' sensory and nutritional qualities can be preserved throughout extended storage by using nanoparticle-based therapies that successfully maintain the fruit's firmness, color, and general quality features. Mango shelf life and quality may be increased by metallic nanoparticles, which may also decrease food waste and increase supply chain effectiveness. However, more investigation, regulatory monitoring, and collaboration are needed.

## 6. Recommendations

The application of metallic nanoparticles as a novel strategy to improve mango postharvest shelf life and quality maintenance has been investigated in a number of studies.

- To maximize the antibacterial, antioxidant, and ripening-delay characteristics and optimize the ratios and concentrations of the nanoparticle combinations
- Undertake thorough toxicological analyses to guarantee that mangoes treated with nanoparticles are safe for ingestion by humans and the environment
- Examine economical and efficient ways to include metallic nanoparticles in the handling and processing of mangoes
- Conduct comprehensive shelf-life assessments in a range of storage environments, incorporating actual supply chain situations, to assess the enduring effectiveness of nanoparticle-based remediation
- Engage customers to find out what they think about the use of metallic nanoparticles in mango fruit preservation

## Figures and Tables

**Table 1:** Using different nanoparticles to extend the shelf life of mango fruit.

Sources of nanoparticles	Synthesis	Characterization	Size of nanoparticle	Shape of nanoparticle	Type of nanoparticle	Method of applying	Mechanism of preservation	References
*Trifolium alexandrinum*	Biological method	FT-IR	49.6 nm		AgNPs	Spray	In addition to having antibacterial properties, AgNPs' bioconjugate also has oxygen-scavenging properties	[60]
Mango leaf	Biological method	UV-Vis, TEM	33.90 nm	Spherical	AgNPs	Coating	Breaks the cell membrane or modifies the protein-lipid bilayer, preventing fungal respiration and fungal metabolism	[61]
*Alpinia officinarum*	Biological method	SEM, XRD, FTIR	—	Amorphous	CuO NPs	Coating	Postponed fruit deterioration and discoloration and decreased weight loss	[62]
Commercial	Chemical method	TEM, FTIR, SEM	245.2 nm	Spherical	ZnO NPs	Coating	Production of reactive oxygen species (ROS), which can disrupt cellular processes and harm bacterial proteins, DNA, and lipids	[63]
Commercial	Chemical method	UV-Vis, TEM, FTIR	5.84 nm to 10.2 nm	Spherical	AgNPs	Coating	Mango fruit's shelf life is prolonged and its ripening is effectively delayed by AgNPs' coatings	[64]
Commercial	Chemical method	WVTR	—	Cylindrical	ZnO	Coating	The growth of *E. coli* is efficiently inhibited by ZnO NPs	[65]
Commercial	Chemical method	SEM			TiO_2_	Coating	Mango fruit deterioration might be more successfully inhibited by TiO_2_	[66]

## Data Availability

During the review process, no new data were generated or examined; instead, individual studies were merged into one to provide the readers with all the information they needed.

## Abbreviations

AgNPs: Silver nanoparticles
CuO-NPs: Copper oxide nanoparticles
TiO_2_-NPs: Titanium oxide nanoparticles
ZnO-NPs: Zinc oxide nanoparticles
ROS: Reactive oxygen species
UV-Vis: Ultraviolet visible
TEM: Transmission electron microscope
SEM: Scanning electron microscope
XRD: X-ray diffraction
WVTR: Water vapor transmission rate.
